# Vector competence of *Culicoides sonorensis* (Diptera: Ceratopogonidae) to epizootic hemorrhagic disease virus serotype 7

**DOI:** 10.1186/1756-3305-5-236

**Published:** 2012-10-17

**Authors:** Elizabeth W Howerth, David E Stallknecht, Deborah L Carter, Barbara S Drolet, Eyal Klement, Daniel G Mead

**Affiliations:** 1 Southeastern Cooperative Wildlife Disease Study, College of Veterinary Medicine, University of Georgia, Athens, GA 30602, USA; 2 Department of Pathology, College of Veterinary Medicine, University of Georgia, Athens, GA 30602, USA; 3 United States Department of Agriculture, Agricultural Research Service, Arthropod-Borne Animal Diseases Research Unit, Manhattan, KS 66502, USA; 4 Koret School of Veterinary Medicine, The Robert H. Smith Faculty of Agriculture, Food, and Environment, The Hebrew University of Jerusalem, P.O. Box 12, Rehovot 76100, Israel; 5 Present address: United States Department of Agriculture, Agricultural Research Service, Arthropod-Borne Animal Diseases Research Unit, Manhattan, KS 66502, USA; 6 Present address: Baker Institute for Animal Health, Department of Microbiology and Immunology, College of Veterinary Medicine, Cornell University, Ithaca, NY 14853, USA

**Keywords:** *Culicoides sonorensis*, EHDV-7, Epizootic hemorrhagic disease, Hemorrhagic disease, Transmission, Vector competence, White-tailed deer

## Abstract

**Background:**

*Culicoides sonorensis* (Diptera: Ceratopogonidae) is a vector of epizootic hemorrhagic disease virus (EHDV) serotypes 1 and 2 in North America, where these viruses are well-known pathogens of white-tailed deer (WTD) and other wild ruminants. Although historically rare, reports of clinical EHDV infection in cattle have increased in some parts of the world over the past decade. In 2006, an EHDV-7 epizootic in cattle resulted in economic loss for the Israeli dairy industry. White-tailed deer are susceptible to EHDV-7 infection and disease; however, this serotype is exotic to the US and the susceptibility of *C. sonorensis* to this cattle-virulent EHDV is not known. The objective of the study was to determine if *C. sonorensis* is susceptible to EHDV-7 infection and is a competent vector.

**Methods:**

To evaluate the susceptibility of *C. sonorensis*, midges were fed on EHDV-7 infected WTD, held at 22 ± 1°C, and processed individually for virus isolation and titration on 4–16 days post feeding (dpf). Midges with a virus titer of ≥10^2.7^ median tissue culture infective doses (TCID_50_)/midge were considered potentially competent. To determine if infected *C. sonorensis* were capable of transmitting EHDV-7 to a host, a susceptible WTD was then fed on by a group of 14–16 dpf midges.

**Results:**

From 4–16 dpf, 45% (156/350) of midges that fed on WTD with high titer viremia (>10^7^ TCID_50_/ml) were virus isolation-positive, and starting from 10–16 dpf, 32% (35/109) of these virus isolation-positive midges were potentially competent (≥10^2.7^ TCID_50_/midge). Midges that fed on infected deer transmitted the virus to a susceptible WTD at 14–16 dpf. The WTD developed viremia and severe clinical disease.

**Conclusion:**

This study demonstrates that *C. sonorensis* is susceptible to EHDV-7 infection and can transmit the virus to susceptible WTD, thus, *C. sonorensis* should be considered a potential vector of EHDV-7. Together with previous work, this study demonstrates that North America has a susceptible ruminant and vector host for this exotic, cattle-virulent strain of EHDV-7.

## Background

The epizootic hemorrhagic disease (EHD) virus (EHDV) serogroup is in the genus *Orbivirus*, family Reoviridae and is comprised of seven proposed serotypes worldwide [1]. Prior to 2006, endemic serotypes in North America included EHDV-1 and -2. However, since 2006, EHDV-6 (strain Indiana), has been repeatedly isolated from white-tailed deer (WTD; *Odocoileus virginianus*) over a wide geographic area in the US and may represent a third endemic serotype [2]. These three EHDV serotypes, along with multiple serotypes of bluetongue virus (BTV) are well-recognized pathogens of WTD and are etiologic agents of hemorrhagic disease (HD), one of the most significant infectious diseases of WTD [3]. Additionally, there are reports of EHDV causing disease in other wild ruminant species, as well as domestic cattle [3]. While infection of cattle with EHDV is not uncommon, clinical disease is generally absent or rarely manifests as a mild bluetongue-like disease [4-6]. One notable exception to this has been multiple reports of clinical disease in cattle associated with EHDV-2 (strain Ibaraki) in East Asia since the 1950’s [7]. Since 2003, however, reports of clinical disease in cattle associated with EHDV have increased in some parts of the world [8-10]. One such report occurred during 2006 in Israel where EHDV-7 caused a widespread and intense epizootic in Israeli cattle, resulting in significant production loss [10,11]. While BTV has been present in Israel for decades, EHDV was not known to be present in Israel prior to the 2006 EHDV-7 outbreak. Although the source of this incursion into Israel remains uncertain, dispersal of infected *Culicoides* biting midges via winds, not animal movement, was determined to have been the primary factor in the spread of the outbreak across northern Israel [12]. Other than this 2006 outbreak, the only other report of EHDV-7 was the original isolation of the virus from a sentinel cow in Australia [13].

Numerous recent events have collectively served to renew interest in some fundamental areas of orbivirus research, including: the reports of EHD in cattle mentioned above; the emergence and rapid range expansion of multiple BTV serotypes throughout northern Europe associated with severe disease in sheep and cattle [14,15]; and the identification of 10 historically non-endemic BTV serotypes [16] and one EHDV serotype [2] in the US. Of particular interest is the risk that introduced exotic viruses may become established in North America, which requires competent vectors and susceptible hosts. To this end, we recently demonstrated that WTD are susceptible to infection with EHDV-7 and that disease is severe and clinically indistinguishable from HD associated with recognized endemic EHDV and BTV serotypes [17]. In the US, *C. sonorensis* is the only confirmed vector for EHDV-1 and -2 [18,19]. Thus, in the current study we evaluated *C. sonorensis* as a potential EHDV-7 vector. Our specific research objectives were 1) to determine if *C. sonorensis* is susceptible to oral infection, and 2) to determine if infected midges can transmit this exotic virus to a susceptible WTD.

## Methods

### *Culicoides* infection

 Laboratory-reared *C. sonorensis*[20] were obtained from each of the three colonies maintained at the Arthropod-Borne Animal Diseases Research Unit (USDA, Manhattan, KS, USA). Pooled 1-day-old male and female midges were shipped overnight to the University of Georgia and were kept in an insectary at 22 ± 1°C on a 12:12 light–dark cycle, and provided 10% sucrose ad libitum. Midges were 3–4 days-old at the time of infection. For infection of *Culicoides*, four, 8-month-old WTD were experimentally infected with EHDV-7 [17]. One additional WTD was maintained in a separate enclosure to use for subsequent insect-to-animal transmission. All procedures were approved by the University of Georgia’s Institutional Animal Care and Use Committee.

**Figure 1:**
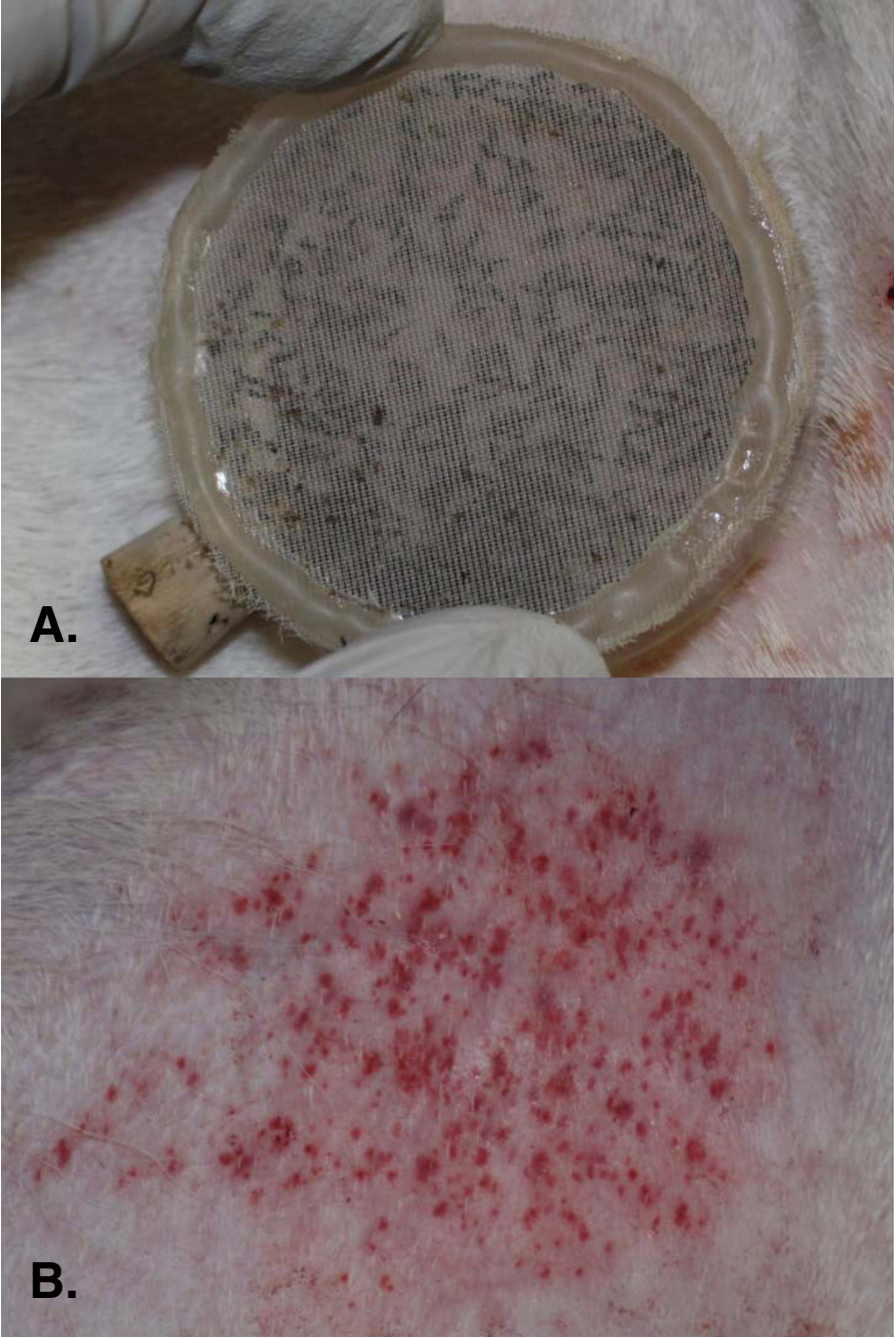
**Feeding of *C. sonorensis *on white-tailed deer.A**) feeding cage containing *C. sonorensis* held firmly against closely shaved skin of a white-tailed deer experimentally infected with EHDV-7. **B**) bite wounds present after approximately 30 minutes of feeding.

In order to coincide with anticipated peak viremia, midge feeding trials were performed on deer at 5–7 days post-infection (dpi). Eight feeding trials were performed on four deer, with midges feeding on three of the WTD on consecutive days. For the feeding trials, midges were briefly anesthetized with CO_2_ and transferred to feeding cages in batches of 150 to 200 individuals (male and female). Cages were made from 5-cm-diameter polyvinyl chloride (PVC) pipe and cut into 1.5-cm tall sections with a fine polyester mesh enclosing each side. Closely shaved regions of the caudoventral abdomen served as feeding sites (Figure 1). The WTD were sedated with xylazine (1–2 mg/kg IM) plus ketamine (2–3 mg/kg IM) and the feeding cages were held firmly against the skin in low light for approximately 30 minutes. After feeding, sedation was reversed with tolazoline (2–4 mg/kg IV slowly). Midges were sorted under a dissecting microscope according to feeding status (i.e., blood fed or non-blood-fed) and five blood fed midges from each feeding trial were placed into individual 1.5-ml microcentrifuge tubes containing 500-μl of virus transport media (minimum essential medium with 10% fetal bovine serum and antibiotics [500 units penicillin, 0.5 mg streptomycin, and 1.25 μg amphotericin B/ml] (Sigma Chemical Company, St. Louis, MO USA) for virus isolation. All remaining blood fed midges were held in an insectary for 4–16 days at 22 ± 1°C, as described above.

### *Culicoides* testing

To determine if *C. sonorensis* midges were susceptible to infection, ≥ 10 were harvested each day beginning 4 days post-feeding (dpf) and continuing through 16 dpf. Cages were checked twice daily for dead or dying insects and any midges that were slow moving or poorly responsive were opportunistically harvested before death. Dead midges were discarded. At 14–16 dpf, surviving midges were transferred to feeding cages and allowed to feed on a naïve WTD, as described above. Midges were immediately sorted according to feeding status and were processed for virus isolation and titration.

### *Culicoides* transmission of EHDV-7 to white-tailed deer

The WTD used to demonstrate insect-to-animal transmission was obtained from the University of Georgia’s Whitehall Deer Research Facility, Whitehall Experimental Forest, Clarke County, Georgia (USA). The animal was hand-raised and housed indoors in an animal biosafety level-2 facility at the University of Georgia. The study occurred during February when wild midges are not present in north Georgia, but as an extra precaution a fine mesh cover was placed over ventilation ducts to prevent insect incursion. The animal was seronegative and virus negative prior to use, as determined by methods described below.

The WTD fed on by the 14–16 dpf midges was monitored two to three times daily for evidence of clinical disease. At approximately the same time each day (−3, 0, 4, 6, 7, and 9 dpf), the challenged deer was sedated for physical examination and blood collected for virus isolation and titration, serology, complete blood count, serum total protein, and coagulation assays [prothrombin time (PT) and activated partial thromboplastin time (APTT)]. All clinical pathology assays were performed using standard protocols by the University of Georgia’s Clinical Pathology Lab (College of Veterinary Medicine, Athens, GA, USA). At the end of the study, the animal was euthanized with sodium pentobarbital (1 ml/5 kg IV), and a complete gross and microscopic postmortem examination was performed. In addition, tissue samples were collected in virus transport media for titration, including cerebrum, cerebellum, heart, lung, spleen, lymph node, skin, and epididymis. Serology was performed using agar gel immunodiffusion tests (AGID; Veterinary Diagnostic Technology, Wheatridge, CO, USA) and serum neutralization as previously described [21].

### Virus isolation and titration

Midges were placed in virus transport media and were held at 4°C for up to 48 hours before being processed individually for virus isolation and titration. For virus isolation, individual midges were manually homogenized using sterile pestles and sonicated for 15 seconds using a sonicating water bath (Branson, Sonic Power Company, Danbury, CT, USA). Homogenized midges were then centrifuged at 4°C for 12 minutes at 1,500 x g. For virus isolation, supernatant (200 μl) was inoculated onto BHK_21_ and cattle pulmonary arterial endothelium cells (CPAE) (American Type Culture Collection, Manassas, VA, USA) in a 24-well format [22]. In addition to virus isolation, all midges were individually titrated for virus using BHK_21_ cells as previously described [22] and endpoint titers (TCID_50_) were determined [23]. Because of the dilutions used, the minimum detectable titer was 10^2.3^ TCID_50_/midge. Based on previous studies in domestic sheep with BTV and *C. sonorensis*, we considered midges with virus titers of ≥10^2.7^ TCID_50_ to be potentially competent, as this was the virus titer of midges capable of efficient transmission to susceptible hosts [24].

All virus isolation and titration attempts from whole blood were performed using CPAE cells as previously described [25], and from tissue samples as previously described [17]. Cell culture supernatant was collected from cultures exhibiting cytopathic effect and RNA extracted using a QIAamp® Viral RNA Mini kit (Qiagen, Valencia, CA, USA) according to manufacturer instructions. Virus isolates were confirmed as EHDV-7 by RT-PCR using previously published primers [26].

### Statistical analysis

For the purpose of statistical analysis, midges were grouped into two categories according to the time elapsed from feeding: the 4–9 dpf group was compared to the 10–16 dpf group for midges fed on WTD with low-titer viremia and high-titer viremia. To statistically compare these values, a Chi-square test was performed. Statistical significance for the increase in the percentage of potentially competent virus-positive midges from 10–16 dpf was performed using the Cochran-Armitage Chi-square test for trend. All analyses were performed using COMPARE2 module in the WINPEPI statistical package [27]. A p-value < 0.05 was considered as indicative for statistical significance in all analyses.

## Results

### Susceptibility to infection

**Figure 2:**
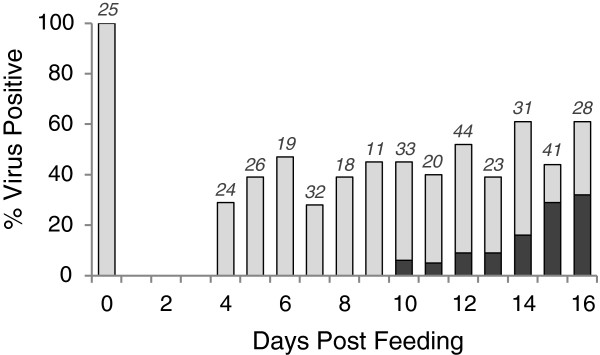
**Bar graph showing replication of EHDV-7 over time in *C. sonorensis *that were infected by feeding on WTD with a high-titer viremia (mean 10**^**7.24**^**TCID**_**50**_**/ml).** The light bars show percent virus isolation-positive midges (no. positive/no. tested x 100), and the dark bars show the percent of virus isolation-positive midges with a high virus titer (no. of midges with ≥ 10^2.7^ TCID_50_ per midge/no. of virus isolation-positive midges x 100). Numbers above bars represent the number of blood fed midges processed at each time point

The level of viremia in the four WTD used to infect the midges varied: two animals had relatively low-titer viremias (10^3.1^ – 10^3.94^ TCID_50_/ml) and two had high-titer viremias (10^7.03^ – 10^7.6^ TCID_50_/ml). There were three feeding trials on the two animals with low-titer viremias and five feeding trials on the two animals with high-titer viremias. Virus isolation and titration results from all midges are presented in Table 1. When provided a high-titer blood meal, *C. sonorensis* were susceptible to oral infection with EHDV-7, with a total virus recovery rate of 36% (47/130) from 4–9 dpf and 50% (109/220) from 10–16 dpf. Replication to titers higher than the competence threshold (≥10^2.7^ TCID_50_/midge) occurred in 32% (35/109) of virus-positive midges between 10–16 dpf, with a mean virus titer of 10^3.21^ TCID_50_/midge. During this time period, there was a statistically significant increase in the percentage of potentially competent virus-positive midges (p=0.001) (Figure 2), with the highest percentage (60%, 21/35) occurring on 15–16 dpf. However, rather than considering only virus positive midges in these calculations, if all blood fed midges between 10–16 dpf (n=220) are included, then 10% (21/220) of blood fed midges were potentially competent. Poor virus recovery rate was observed from midges that were fed on WTD with low-titer viremias, both at 4–9 dpf (5%; 2/37) and 10–16 dpf (4%, 4/93), and none of these virus isolation-positive midges had a high virus titer (Table 1). The difference in virus recovery rates between 4–9 dpf and 10–16 dpf midges fed on WTD with high-titer viremia versus those fed on WTD with low-titer viremia were highly significant (p<0.0001).

**Table 1:** **Virus isolation and titration data from *Culicoides sonorensis *infected with EHDV-7 after feeding on white-tailed deer with either a low-titer or high-titer viremia**

	**Low-titer viremia**^***a***^		**High-titer viremia**^***b***^	
**dpf**^***c***^	**% VI positive (no. positive/n)**	**Virus titer per midge**^***d***^**(n)**	**% VI positive (no. positive/n)**	**Virus titer per midge**^***d***^**(n)**
0	33 (5/15)	<2.3(5)	100 (25/25)	<2.3(18), 2.3(4), 2.5 (3)
4	0 (0/8)		29 (7/24)	<2.3(7)
5	0 (0/3)		39 (10/26)	<2.3(10)
6	0 (0/2)		47 (9/19)	<2.3(7), 2.3(2)
7	5 (1/21)	<2.3(1)	28 (9/32)	<2.3(9)
8			39 (7/18)	<2.3(7)
9	33 (1/3)	<2.3(1)	45 (5/11)	<2.3(5)
10	0 (0/1)		45 (15/33)	<2.3(11), 2.3, 2.63, 2.97, 3.3
11	0 (0/1)		40 (8/20)	<2.3(7), 3.1
12	10 (2/21)	<2.3(2)	52 (23/44)	<2.3(14), 2.3 (3), 2.5 (2), 2.73 (3), 3.8
13	3 (1/35)	<2.3 (1)	39 (9/23)	<2.3 (3), 2.63 (4), 2.97, 3.63
14	0 (0/14)		61 (19/31)	<2.3(13), 2.63, 2.73, 2.8, 2.97, 3.1, 3.8
15			44 (18/41)	<2.3(6), 2.8 (2), 2.87 (2), 2.97 (2), 3.1 (2), 3.63, 3.73, 3.8, 3.87
16	5 (1/21)	<2.3(1)	61 (17/28)	<2.3(5), 2.5 (2), 2.63, 2.73, 3.1 (2), 3.3 (2), 3.63, 3.8, 4.1, 4.63
Total^*e*^	5 (6/130)		45 (156/350)	

^*a*^ viremic WTD had a mean virus titer of 10^3.59^ TCID_50_/ml (range, 10^3.1^- 10^3.9^ TCID_50_/ml).

^*b*^ viremic WTD had a mean virus titer of 10^7.24^ TCID_50_/ml (range from 10^7.03^- 10^7.6^ TCID_50_/ml).

^*c*^ days post-feeding.

^*d*^ log_10_ TCID_50_/midge.

^*e*^ includes data from day 4 – 16 dpf.

### Vector competence

**Figure 3:**
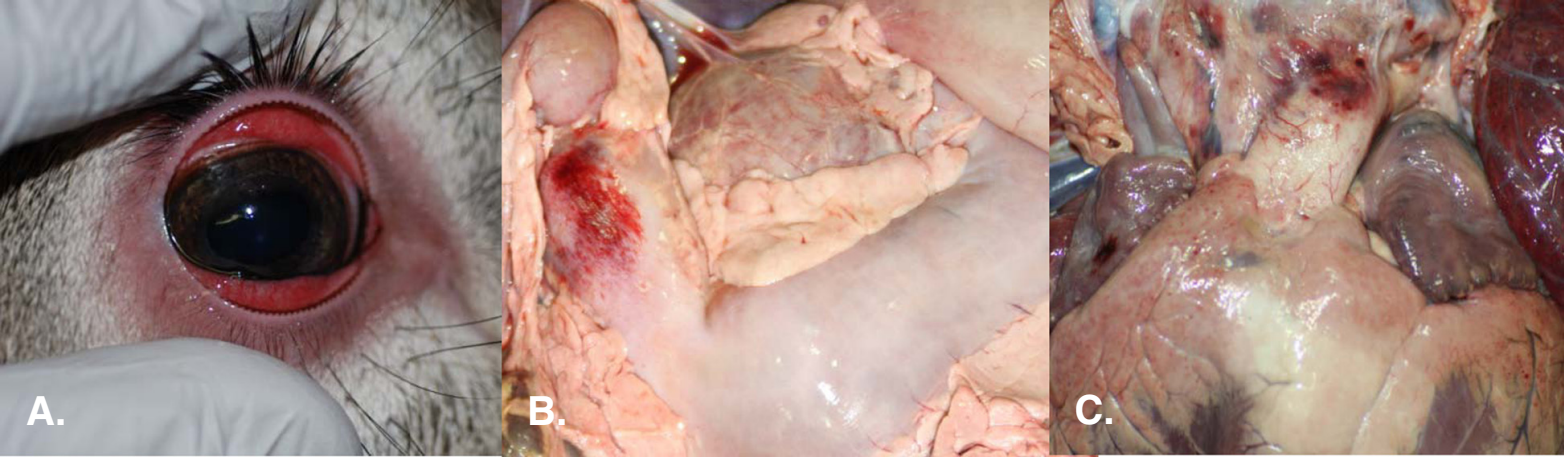
**Clinical outcome of a white-tailed deer infected with EHDV-7 by the bite of *C. sonorensis. *A**) Photograph taken on 9 dpf just prior to euthanasia showing prominent erythema of periorbital skin and severe congestion and edema of the conjunctiva. **B**) Paintbrush hemorrhage in the wall of the abomasum at the level of the pylorus. **C**) Subintimal hemorrhage at the base of the pulmonary artery. [Both **B**) and **C**) are considered classic lesions of acute hemorrhagic disease in WTD.].

Of midges that originally fed on WTD with high titer viremia, 100 survived to 14–16 dpf. Before processing for virus isolation and titration, these midges were given the opportunity to take a blood meal from a naïve WTD in order to attempt transmission. Midges were sorted according to feeding status immediately after the feeding attempt and only 44 of the 100 midges (44%) had taken a blood meal. Virus isolation and titration results indicated that 27 of these 44 blood fed midges (61%) were infected (i.e., virus isolation positive) at the time of feeding. Of these 27 infected midges, titration results indicated that 12 (44%) had virus titers ≥ 10^2.7^ TCID_50_/midge, with a geometric mean titer of 10^3.51^ TCID_50_/midge (range: 10^2.73^ - 10^4.63^ TCID_50_/midge). However, rather than considering only virus-positive midges in the above percentages, 12 % (12/100) of the 100 midges allowed to take a blood meal were potentially competent. Midge-to-deer transmission was demonstrated, as evidenced by a detectable viremia in the deer by 4 dpf, which peaked on 6 dpf. The viremia profile, clinical pathology findings, and rectal temperature from the WTD are presented in Table 2. The animal exhibited clinical signs consistent with HD, such as fever, dull-coat, lethargy, decreased appetite, depression, erythema of non-haired skin, and hyperemia with edema of conjunctiva (Figure 3A). By 9 dpf, the animal had to be euthanized due to abundant hemorrhages in the oral mucosa, severe bleeding tendencies, and a progression of the lethargy and depression.

**Table 2:** **Summary of the viremia profile, hematology, coagulation assays, and rectal temperature abnormalities of a white-tailed deer infected by blood feeding EHDV-7-infected *C. sonorensis *midges**

		**Days Post Feeding**					
**Test**	**Units**	**−3**	**0**	**4**	**6**	**7**	**9**
Viremia	*log*_*10*_*TCID*_*50*_*/ml*	‐^*a*^	‐	6.4	7.27*^b^	7.1	5.26
Total WBC^*c*^	*x cells/μl*	1,700	2,000	nd^*d*^	600*	700	2,100
Lymphocytes	*x cells/μl*	714	760	nd	90*	91	378
Hematocrit	*%*	38.2	38.6	nd	42.5	42.4	49.1*
Total protein	*g/dl*	5.4	5.5	nd	4.7	5.5	3.8*
PT^*e*^	*seconds*	13.2	12.6	12.4	nd	17	19*
APTT^*f*^	*seconds*	23.5	20.8	22.4	nd	35.7	51*
Platelets	*x 10*^*3*^	725	491	nd	356	266	47*
Rectal temp.	*° F*	101.5	101	101.4	104.6*	103.1	99.8

^*a*^ negative virus isolation.

^*b*^ asterisk indicates the peak abnormality in test.

^*c*^ total white blood cell count.

^*d*^ not done.

^*e*^ prothrombin time.

^*f*^ activated partial thromboplastin time.

 Postmortem examination revealed widespread congestion and hemorrhage in a variety of tissues. Hemorrhages were common in the oral cavity, including the dental pad, palatal ridges, and buccal papillae. Bilateral, severe, diffuse, epididymal hemorrhages were noted, with multiple small foci of hemorrhage within the testicular parenchyma. Additionally, a subadventitial hemorrhage at the base of the pulmonary artery and a paintbrush hemorrhage within the wall of the pylorus (Figure 3B and C) were observed. These are considered classic lesions of acute HD in this species. Virus was isolated from all tissues sampled, though virus titers (TCID_50_/g) were variable: cerebrum, 10^4.03^; cerebellum, 10^3.6^; heart, <10^3.6^; lung, 10^5.94^; spleen, 10^6.03^; lymph node, 10^5.1^; skin, 10^5.8^; epididymis, 10^5.8^. Precipitating and neutralizing antibodies were detected by 9 dpf.

## Discussion

Our findings demonstrate that *C. sonorensis* is a potential competent vector for EHDV-7, as evidenced by the ability of the midge to become infected following ingestion of a blood meal from an experimentally infected WTD and subsequent bite transmission of EHDV-7 from *C. sonorensis* to a naive WTD. The observed viral dynamics, clinical abnormalities, and postmortem findings in the challenge animal were consistent with previous reports of HD in WTD, as reviewed by Howerth *et al*. [3]. Four days after feeding infected midges on the naïve WTD, the animal had a high-titer viremia and the exhibited clinical signs and the incubation period were consistent with HD in this species [28,29]. Lymphopenia, erythrocytosis, hypoproteinemia, thrombocytopenia, and prolongation of PT and APTT were observed, all of which are common abnormalities in WTD infected with EHDV and/or BTV [25,29]. The coagulopathy in this animal was severe, based on both the clinical pathology findings, as well as clinical observation of bleeding tendencies at the time of euthanasia on 9 dpf.

Only 27 of the 44 midges that took a blood meal from the naïve deer were found to be infected at the time of feeding, thus it may be assumed that these 27 midges were responsible for the EHDV transmission. However, based on the titration data, only a portion of infected midges were likely to be competent (12/27, 44%). Thus, it is suspected that one or more of these 12 midges were responsible for transmission. While only 44 of 100 midges had a visible blood meal after the transmission attempt, we cannot rule out the possibility of probing without blood feeding by any of the 14 other potentially competent midges, which could have potentially contributed to virus transmission. The efficiency of EHDV transmission to WTD by *C. sonorensis* has not been investigated, nor is a minimum infectious dose known. The bite from a single infected *C. sonorensis* has been shown to transmit BTV-1 to domestic sheep [30,31] and similar studies are necessary to better understand virus transmission to WTD.

Our results suggest that, for EHDV-7, infection of blood fed midges is less efficient (4.6%, 6/130) when viremia was < 10^4.0^ TCID_50_/ml. This lower infection rate is not surprising, considering the poor virus recovery rate on 0 dpf (33%; 5/15) when midges were provided a lower-titer blood meal (Table 1). A previous experimental infection of *C. sonorensis* with EHDV encountered a similar low infection rate when the blood meal titer ranged from 10^2.1^ – 10^3.0^ TCID_50_/ml [22]. Our study did not examine an infection threshold, but the finding is interesting and highlights our lack of understanding of some very fundamental factors regarding the transmission of EHDV. Additionally, we do not know for how long a viremic WTD is infectious to *Culicoides*, specifically when viremia is of low titer (<10^2.3^ TCID_50_/ml). It is known that WTD experimentally infected with EHDV can be viremic for as long as 56 days post-inoculation [25]; however, only transiently is the viremia comparable to the peak titers in our study (>10^7.0^ TCID_50_/ml) which resulted in high infection rates in the midges. Previous studies show that peak viremia in WTD generally occurs 5–7 days post inoculation and blood virus titers of 10^5.0–6.0^ TCID_50_/ml may be present for as long as seven days, whereas titers > 10^6.0^ TCID_50_/ml are generally only present for 2–3 days early in the infection [25,32]. After the appearance of neutralizing antibodies the blood virus titer generally declines sharply 8 to 12 dpi. Future studies should examine the epidemiological significance of the prolonged low-titer viremia in WTD. Although the efficiency of transmission from these animals to *Culicoides* has not been explored, it is important to consider additional factors regarding transmission efficiency. For instance, the attack rate on WTD by *Culicoides* midges can be remarkably high. In a study by Smith and others, >10,000 *C. debilipalpis* (formerly *lahillei*) were aspirated from a WTD on five of six autumn mornings, with a maximum single collection of 20,840 midges [33].

The World Health Organization (WHO) has established criteria for the designation of an arthropod as a confirmed vector of an arbovirus: 1) recovery of virus from non-blood-fed wild-caught specimens, 2) demonstration of the ability to become infected experimentally, 3) demonstration of biological transmission, and 4) an accumulation of field evidence confirming an association of the infected arthropods and the appropriate vertebrate host [34]. This study has fulfilled the second and third WHO criteria, thus, we conclude that *C. sonorensis* should be considered a potential vector of EHDV-7. Since EHDV-7 is exotic to North America, criteria 1 cannot be fulfilled. Although field research is scarce, previous studies have provided some evidence for both criteria 1 [18,35,36] and 4 [18], as they relate to *C. sonorensis* serving as a vector of EHDV serotypes endemic to the US for WTD.

Although there is significant field evidence to show that *C. sonorensis* is the primary vector of BTV for sheep and cattle throughout most of the US, the evidence is not as strong for EHDV and BTV transmission to WTD in wild habitat. With the exception of a previous study in Kentucky, where *C. sonorensis* was the predominate species trapped in captive WTD pens during an HD outbreak [19], *C. sonorensis* has been absent or present in very low numbers in subsequent field studies that have been performed in WTD habitat or on captive WTD facilities, using WTD as bait [33,37-39]. In fact, most field studies directly related to WTD suggest that species other than *C. sonorensis* are likely to also be involved in EHDV and BTV transmission to WTD, specifically in the southeastern US. Based primarily on midge abundance, seasonal occurrence, and host preference, it has been suggested that *C. debilipalpis*, *C. stellifer*, *C. obsoletus*, *C. paraensis*, and *C. spinosus* warrant further investigation as potential vectors of EHDV and BTV to WTD in the southeastern US [33,37,39] and *C. debilipalpis* has been shown experimentally to support replication of EHDV-2 [22]. Furthermore, multiple *Culicoides* spp. other than *C. sonorensis* and *C. insignis,* both confirmed BTV vectors, have been listed as suspect or potential vectors of BTV to cattle and sheep in the southeastern US [40]. The above mentioned studies highlight the gaps in our knowledge of the transmission of EHDV and BTV among free-ranging WTD in certain parts of the US where EHDV and BTV are enzootic.

Despite gaps in our knowledge regarding EHDV and BTV transmission among wild ruminants in some portions of the US, *C. sonorensis* was chosen in this study for multiple reasons: 1) it represents the only confirmed vector of EHDV in North America, 2) this species is widely distributed throughout much of the US and commonly feeds on domestic ruminants, thus its competence represents a large geographic risk for outbreaks and establishment following an introduction, and 3) *C. sonorensis* has been colonized, making it possible to test large numbers of insects that consistently feed in captivity.

## Conclusions

Results from this study and previous work [17] demonstrate that North America has both a competent vector and suitable ruminant host for EHDV-7. However, whether EHDV-7 could become established in North America following an introduction depends not only on the mere presence of susceptible ruminant and vector hosts, but also on abiotic factors that can impact vector competence and/or capacity. Climatic variables can impact the *Culicoides* life cycle (particularly midge size and survival), virus replication within the midge, midge behavior, and midge distribution [41]. Although predictions of such introductions and potential establishments are difficult to make, the recent examples of incursions of EHDV-7 in Israel [10], multiple BTV serotypes throughout northern Europe [14], and previously non-endemic BTV serotypes into the US [16,42] clearly demonstrate that the biological and environmental components and restrictions of certain orbivirus episystems are not as stable as previously thought [43].

## Competing interests

The authors declare that they have no competing interests.

## Authors’ contributions

MGR, DGM, DES, and EWH conceived and designed the experiments. MGR, DLC, ABA, and EWH conducted the experimental work. EK and MGR analyzed the data. MGR, DGM, DES, EWH, ABA, BSD, and EK contributed to the manuscript. All authors approved the final version for submission.
